# Spontaneous Regression of Cervical Low-Grade Squamous Intraepithelial Lesions in the Northern Thai Population: Impact of Human Immunodeficiency Virus Infection on Regression Rates and Predictors

**DOI:** 10.3390/jcm14051726

**Published:** 2025-03-04

**Authors:** Anchalee Chainual, Kijja Jearwattanakanok, Jiraporn Khorana, Kittipat Charoenkwan

**Affiliations:** 1 Department of Obstetrics and Gynecology, Nakornping Hospital, Chiang Mai 50180, Thailand; cnanchalee@gmail.com; 2 Department of Surgery, Nakornping Hospital, Chiang Mai 50180, Thailand; jkijja@gmail.com; 3 Department of Surgery, Faculty of Medicine, Chiang Mai University, Chiang Mai 50200, Thailand; nanji22@gmail.com; 4 Department of Biomedical Informatics and Clinical Epidemiology, Faculty of Medicine, Chiang Mai University, Chiang Mai 50200, Thailand; 5 Clinical Surgical Research Center, Department of Surgery, Faculty of Medicine, Chiang Mai University, Chiang Mai 50200, Thailand; 6 Department of Obstetrics and Gynecology, Faculty of Medicine, Chiang Mai University, Chiang Mai 50200, Thailand

**Keywords:** CIN1, HIV, low-grade squamous intraepithelial lesion, LSIL, spontaneous regression

## Abstract

**Background/Objectives**: Low-grade squamous intraepithelial lesions (LSILs) of the cervix are known to have the ability to regress spontaneously. However, in cases where the patient is human immunodeficiency virus (HIV)-positive and has a weakened immune system, the ability to eliminate abnormal cells from the cervix may be impaired. The aim of this study was to determine whether there is an association between the spontaneous regression of histological LSIL and the HIV status of the patient by evaluating baseline characteristics and CD4 count. **Methods**: Women with a diagnosis of cervical histological LSIL were included. We analyzed the correlation between a group of women with LSIL who experienced complete spontaneous regression and those who did not regress based on factors such as HIV status, basic characteristics, and baseline Pap smear. As part of the surveillance program, all the women underwent a Papanicolaou (Pap) smear test every 6 months. **Results**: A total of 127 women were evaluated. The results showed that a higher percentage of women with HIV belonged to the non-regression group compared to the complete regression group ((51.35% vs. 26.67%) *p* = 0.007). After controlling for other factors, the multivariable analysis revealed that HIV-negative women were more likely to experience spontaneous regression of cervical LSIL than women with HIV [HR = 2.54, 95% confidence interval 1.31–4.49, *p* = 0.006)]. **Conclusions**: Cervical histological LSIL had a lower capacity for spontaneous regression in women with HIV. For women who wish to lower their risk of persistent or worsening disease associated with their HIV status, it may be beneficial to undergo active surveillance coupled with additional active treatment or surgery. A CD4 count of over 500 cells per μL is associated with the spontaneous regression of LSIL in women with HIV.

## 1. Introduction

Cervical cancer is the second most common cancer in Thai women. Histological low-grade squamous intraepithelial lesion (LSIL) is considered a relatively benign diagnosis as it often results from a transient expression of human papillomavirus (HPV) infection, which has a low chance of progression and a high rate of spontaneous regression, especially in young women. Histological high-grade squamous intraepithelial lesion (HSIL), on the other hand, is considered a more aggressive pre-cancer that requires prompt intervention [1,2]. The risk of progression from histological LSIL to HSIL and invasive carcinoma is not high; only 11% of untreated LSIL cases eventually progress to HSIL and invasive carcinoma over several decades. This long premalignant period and significant chance of spontaneous regression allow for preventive interventions like the curative excision of the affected area of the cervix in cases with progression to HSIL [3], or observational management for women with histopathological diagnosis of LSIL [4,5,6].

However, human immunodeficiency virus (HIV) infection could increase the risk of HSIL in the cervix. The research gap lies in the uncertainty surrounding LSIL regression in women with HIV and whether conservative management (close follow-up) is sufficient for these cases [7,8]. Therefore, this study was conducted to evaluate the risk of non-regression of histological LSIL in women with HIV and to determine whether the implementation of conservative management by close follow-up is an adequate treatment for histological LSIL in women with HIV.

## 2. Materials and Methods

The study was conducted as a retrospective cohort study. The Research Ethics Committee of Nakornping Hospital approved the study protocol and the waiver of informed consent for retrospective data, with participants identified only by their study number (protocol code 015/64, approved on 11 January 2021).

### 2.1. Participants

Data were collected by reviewing the medical records of women diagnosed with cervical histological LSIL through colposcopy-directed biopsy (CDB) at the colposcopy clinic of our hospital from October 2015 to September 2021. These women were then closely followed up with a surveillance program, which included a repeat Papanicolaou (Pap) smear every six months for a total of four visits during a two-year period.

The exclusion criteria included women with prior histologically confirmed squamous intraepithelial lesion (SIL) or cervical cancer, women who had concomitant histological HSIL, and women who had a history of cervical excision procedures due to cervical dysplasia.

### 2.2. Outcome

Women with four normal Pap smear results in a close follow-up program were classified as the complete regression group. Those who had at least one abnormal Pap smear result were subjected to repeat colposcopy and biopsy at abnormal findings to confirm with pathologic results. If the pathological tissue results confirmed LSIL or worse, they would be identified as a non-regression or persistent group. However, those with an abnormal Pap smear and biopsy results that are less severe than LSIL would be followed according to the protocol and would be considered to have complete regression if no further biopsy-proven LSIL or worse lesions were identified. 

### 2.3. Predictors

The study analyzed the characteristics and risk factors of cervical cancer that might be associated with regression status, including HIV status, age, parity, age of first sexual intercourse (SI), contraception methods, and baseline Pap smear results. All those predictors were analyzed and adjusted for potential multicollinearity during the statistical analysis. This study was particularly interested in HIV status, while the other factors played a confounding role.

### 2.4. Sample Size

The sample size of this study was determined based on data from a pilot study conducted at our hospital during 2015–2016. The pilot study involved 30 cases of cervical histological LSIL, with complete regression (CR) observed in 63.33% of the cases. Within this pilot group, women with HIV showed complete regression in 32% of the cases, whereas HIV-negative women showed complete regression in 68% of the cases. The sample size was estimated with a two-sided test based on regression status and HIV infection status, with a 5% absolute precision and 95% confidence level, and with the ratio of the non-regression group to the complete regression group set at 1:2. As a result, the total sample size was calculated to be at least 66 women with histological LSIL.

### 2.5. Statistical Analysis

Statistical analysis was conducted using the STATA Statistical Package (StataCorp LP, College Station, TX, USA). Descriptive data were presented by count and percentage for categorical data and mean and standard deviation for continuous data. Fisher’s exact test was used for the hypothesis testing of categorical outcome variables, while Student’s *t*-test was used for the hypothesis testing of continuous outcome variables. The significance level for this study was set at 0.05. Multivariable Cox’s regression analysis was used to measure and analyze the association between the patient’s characteristics and the regression status of the histological LSIL. The results were reported using the hazard ratio and the Kaplan–Meier curve.

## 3. Results

**Figure 1:**
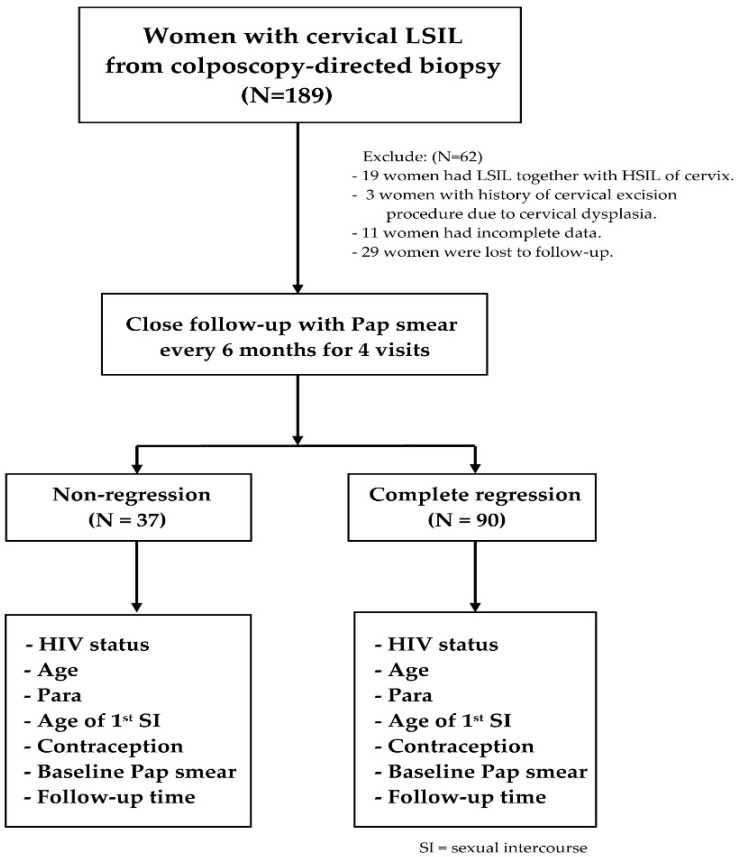
Study flow diagram.

During the period of this study, 189 women with histologically confirmed LSIL identified through colposcopy-directed cervical biopsy were scheduled to follow up at our hospital. Sixty-two women were excluded from the study because they either had a previous cervical excision procedure, co-existing histological HSIL, incomplete data, or lost to follow-up. Of the 127 women included in this study, 90 experienced complete regression, while 37 did not show regression (Figure 1).

Table 1 presents the characteristics of the study cohort stratified by regression status. None of these women were current smokers. A statistically significant difference in HIV status was observed between the two groups. The proportion of HIV-positive women was significantly higher in the non-regression group compared to the complete regression group, 51.35% vs. 26.67%. Most women in the entire cohort had their first sexual intercourse before they turned 30 (92.91%) and did not use contraception (53.54%). Most of the women included in this study had cytological LSIL in the baseline Pap smear, 75.68% in the non-regression group and 67.78% in the complete regression group.

**Figure 2:**
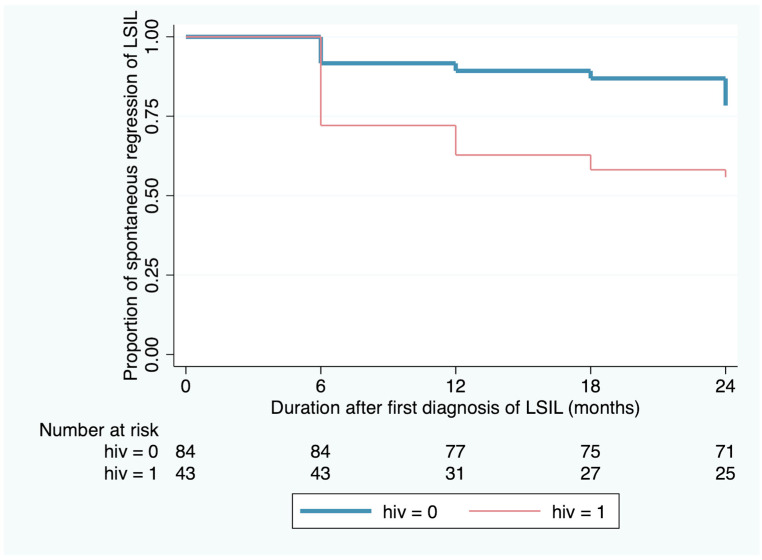
Kaplan–Meier survival curve illustrating the effect of HIV status on the duration of spontaneous regression of low-grade squamous intraepithelial lesions (LSILs).

**Figure 3:**
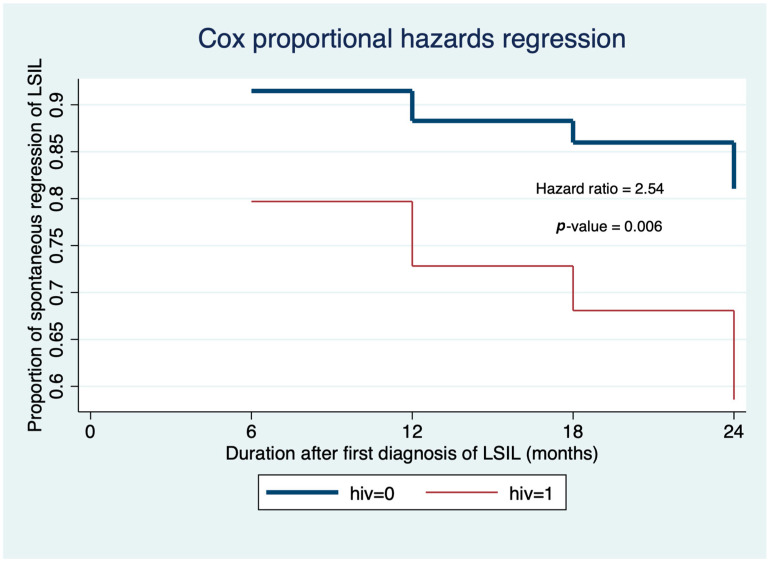
Cox proportional hazard regression curve of the effect of HIV status on the regression status of low-grade squamous intraepithelial lesions (LSILs).

Univariable and multivariable analyses were performed to identify the effect of various factors on the regression of histological LSIL (Table 2). After adjusting for the effects of age, age at first sexual intercourse, parity, contraception use, and baseline Pap smear, we found that the HIV-negative women had a significantly higher chance of regression of histological LSIL of the cervix (multivariable hazard ratio = 2.54, 95% confidence interval 1.31–4.49, *p* = 0.006). The Kaplan–Meier survival curve illustrating the effect of HIV status on the time to diagnosis of persistent histological LSIL and the Cox proportional hazards regression curve demonstrating the effect of HIV status on the regression status of histological LSIL are represented in Figure 2 and Figure 3, where the proportional hazards assumption was satisfied for Cox regression. In the multivariable analysis, we found no significant association between the other factors and histological LSIL regression.

Of the 37 women with non-regression of cervical histological LSIL, most were found to have persistent histological LSIL during the first six months of the follow-up program. Out of the non-regression group, 19 women were HIV-positive, while 18 women were HIV-negative. Among the HIV-positive non-regression group, 11 women had persistence of LSIL, 7 women progressed to HSIL, and 1 woman was found to have early-stage cervical cancer as confirmed by histology. Around one-third of the HIV-positive women with persistent LSIL chose to continue with active surveillance, while 15.97% opted for a loop electrosurgical excision procedure (LEEP). One woman (5.26%) in the HIV-positive group, who had persistent LSIL, decided to undergo hysterectomy due to cancer phobia and dysmenorrhea. The remaining seven women (36.84%) in the HIV-positive group who progressed to HSIL preferred LEEP for treatment and another woman in the HIV-positive group who was diagnosed with early-stage cervical cancer at 12 months of follow-up period received appropriate surgery. Of the 18 women in the HIV-negative non-regression group, 14 (77.78%) had persistent histological LSIL. Of them, nine women (50.00%) were satisfied with continuing follow-up cervical cytology, while the remaining four women (22.22%) preferred excisional procedures and one woman (5.56%) decided to undergo hysterectomy due to cancer phobia and adenomyosis. Four women (22.22%) in the HIV-negative group developed histological HSIL, and they were managed with LEEP according to our treatment protocol for HSIL (Table 3).

The records of CD4 counts within a year before or after the histological diagnosis of LSIL were available for 37 of 43 patients with HIV seropositivity (Table 4). Mean CD4 counts in the complete regression group were significantly higher than the non-regression group (589.95 cells/μL in the complete regression group vs. 360.27 cells/μL in the non-regression group, *p* = 0.004). We observed a higher proportion of patients with CD4 counts of less than 500 cells/μL in the non-regression group compared to the complete regression group, 73.33% vs. 40.91%. The difference was close to a statistically significant level (*p* = 0.053).

## 4. Discussion

It has been found that most histological LSILs of the cervix tend to be transient lesions and have a high incidence of regression. Thus, the guidelines for histological LSIL recommend conservative management with close follow-up and observation. Excisional procedures or ablation are rarely suggested for women who have histological LSIL despite unsatisfactory colposcopy [9]. The American Society for Colposcopy and Cervical Pathology (ASCCP) guideline 2019 recommends that the cervical histological LSIL of the cervix, which is confirmed by tissue pathology, is appropriate for conservative treatment and follow-up for 2 years. Treatments such as excision procedures or ablation may be considered if such a lesion persists for longer than 2 years [4].

Our study has shown that women with the histological LSIL of the cervix could regress spontaneously. The HIV-negative women had a higher rate of complete regression within two years compared to the HIV-positive women. Therefore, our data suggested that HIV infection affects the regression ability of cervical histological LSIL. Many conditions have been recognized as factors that increase the risk of cervical malignancy, including young age at first sexual intercourse, high parity, multiple sexual partners, immunocompromise, HIV status, long-term use of oral contraceptive pills, and history of genital warts [10,11,12]. Our analysis found no correlation between age, age of first sexual intercourse, contraception use, or baseline Pap smear and regression of cervical histological LSIL in both the univariable and multivariable analyses. We included women starting at age 30, consistent with the Ministry of Public Health of Thailand’s recommendation to perform cervical cancer screening in women between the ages of 30 and 60 [13].

Studies suggest that HIV-positive women are at a higher risk of developing pre-cancerous cervical lesions due to greater susceptibility to human papillomavirus (HPV) infection. These lesions are also more likely to persist and progress to higher-grade lesions or cervical cancer compared to HIV-negative women [14,15,16,17,18,19]. A study by Zeier et al. indicated that cervical LSIL could regress spontaneously in women with HIV (36.20%, while in our study, it was 26.67%). They found that while the chance of LSIL progression to higher-grade cervical lesions was the same for both HIV-positive and -negative women, the former experienced more persistent histological LSIL. The excision procedure of the cervix with histological LSIL decreased the risk of progression to higher-grade lesions of the cervix from 54.7% to 0.0% in HIV-negative women and from 46.9% to 6.4% in women with HIV. It also significantly reduced the persistence of histological LSIL in both HIV-negative and -positive women [20]. Other studies by Lima et al. and Omar et al. supported these findings, showing that cervical histological LSIL had poor regression capacity in HIV-positive women, with a higher recurrence rate, positive excisional margins, and glandular involvement. In a prospective cohort study by Lima et al., HIV-positive women had a higher incidence of histological LSIL compared to HIV-negative women. They also found that the recurrence rate of histological LSIL, positive excisional margins, and lesions with glandular involvement was higher in women with HIV [21]. Similarly, Omar et al. found that women with HIV had higher prevalence and incidence of histological HSIL and LSIL, with a relatively low rate of regression from LSIL to normal [22]. Nevertheless, there are conflicting reports about the risk of progression of cervical histological LSIL in HIV-positive women as compared to HIV-negative women. Some studies suggest that there is a higher risk, while others argue that the risk is unclear. However, it is widely recommended that histological LSIL in women with HIV could be treated with observational management similar to HIV-negative women [23,24]. Treatment is only recommended for those who have cervical histological HSIL or higher-grade lesions, which can be treated with excisional procedures [25].

In this study, when only patients with no regression of histological LSIL were considered, we found no significant difference between the HIV-positive and HIV-negative patients in terms of the rate of persistence of LSIL and the rate of progression to HSIL (Table 3). Apart from the HIV status, the timing of the detection could influence the observed severity of these histological abnormalities. Evidence suggests that active treatments are helpful in women with HIV with the non-regression of cervical intraepithelial lesions. Cryotherapy for cervical histological LSIL is found to reduce the risk of progression to histological HSIL among women with HIV in limited resource situations [26]. The excisional procedure is also effective for treating cervical dysplasia in women with HIV, and it is not significantly related to complications when compared with HIV-negative women [27]. In this study, about two-thirds (7 out of 11) of the women with HIV with persistent cervical LSIL decided to continue the surveillance program, while one-third underwent excisional procedures like LEEP or hysterectomy. Per our management protocol, all the women with disease progression to HSIL, regardless of their HIV status, were offered excisional procedures, mainly LEEP. Studies have also found that the recurrence of cervical intraepithelial lesions after treatment with excisional procedures is more common in women with HIV. Dysplastic cells are often found at the excisional margin, which is why excisional treatment with a completely clear margin status is suggested in women with HIV who need excisional treatment. Close follow-up is recommended, especially for women with positive excisional margin status [28,29,30]. This recommendation should also be emphasized in women with HIV with persistent LSIL who choose to remain in the surveillance program without active treatment.

This study has certain limitations that need to be considered. Firstly, it was a retrospective study that reviewed the data from out-patient records, which resulted in some missing related information that might introduce selection bias and not be completely representative of the population. In addition, the small sample size resulted in a limited number of patients in certain categories of the nominal risk factors, such as parity, age at first sexual intercourse, contraception, and baseline Pap results. This could potentially restrict the ability to detect any association between these factors and LSIL regression. Finally, due to its retrospective nature, it was difficult to completely evaluate the HIV disease control of the subjects, even though this is an important aspect. As some studies have shown, HIV control with antiviral agents, CD4 counts, and HIV viral load are factors that affect HPV infection and outcomes of cervical dysplasia treatments [15,28,31,32].

When evaluating the association between CD4 counts and LSIL regression in patients with HIV, we found that the mean CD4 count in the non-regression group was significantly lower than that observed in the complete regression group. This finding is consistent with studies that claim that the incidence of developing cervical intraepithelial lesions and the risk of progression to higher-grade cervical lesions in HIV-positive women with CD4 counts over 500 cells/μL are similar to those in HIV-negative women [22,33]. Additionally, we identified a higher percentage of patients with CD4 counts below 500 cells/μL in the non-regression group compared to the complete regression group, 73.33% versus 40.09%. Although the difference was not statistically significant, with a *p*-value of 0.053 for CD4 categories slightly exceeding the conventional threshold of 0.05, this may stem from limited power due to a small sample size. Nevertheless, the trend indicates that a CD4 count below 500 could be associated with a lower likelihood of spontaneous LSIL regression. Therefore, further prospective assessment of the management of histological LSIL and HIV disease control would be beneficial.

Our study found that women with a parity of three had significantly higher rates of LSIL regression, which was somewhat unexpected. Previous research has suggested that higher parity may act as a co-factor in the progression from HPV infection to cervical cancer, potentially increasing the risk of persistent HPV infection [34,35,36]. However, this discrepancy may be due to selection bias, as we experienced a significant number of follow-up case losses. Additionally, our sample size might not be large enough to accurately capture the expected relationship between parity and the risk of persistent cervical dysplasia. A larger study population could help determine whether our findings represent a genuine biological effect or are simply the result of random variation.

## 5. Conclusions

In conclusion, cervical histological LSIL is less likely to regress spontaneously in women with HIV. Minimally invasive excisional procedures, such as LEEP or conventional conization, should be carefully considered or included in practice guidelines for this particular group of patients. Higher CD4 counts, particularly those above 500, appear to support the spontaneous regression of cervical LSIL in women with HIV, which could inform those who prefer conservative treatment for cervical dysplasia in this population. This study found an unexpected correlation between higher parity and a higher regression rate of LSIL, which should be further studied to establish accurate information.

## Figures and Tables

**Table 1:** Participants’ characteristics according to regression status.

Characteristics	Non-Regression (N = 37)		Complete Regression (N = 90)		*p*-Value
	N	%	N	%	
HIV status					
HIV-Positive	19	51.35	24	26.67	0.007 *
HIV-Negative	18	48.65	66	73.33	
Age, years [mean ± SD]	38.19 ± 9.15		39.08 ± 10.49		0.654
<30 years	8	21.62	17	18.89	0.652
30–59 years	28	75.68	72	80.00	
≥60 years	1	2.70	1	1.11	
Parity					0.061
0	14	37.84	19	21.11	
1	11	29.73	29	32.22	
2	8	21.62	24	26.67	
3	1	2.70	15	16.67	
≥4	3	8.11	3	3.33	
Age of 1st SI, years. (mean ± SD)	20.84 (±5.73)		20.27 (±4.42)		0.546
<20	20	54.05	41	45.56	0.376
21–29	13	35.14	44	48.89	
30–39	3	8.11	4	4.44	
≥40	1	2.70	1	1.11	
Contraception					0.111
No contraception	22	59.46	46	51.11	
Oral contraceptive pills	1	2.70	16	17.78	
Depo-medroxyprogesterone	1	2.70	7	7.78	
acetate					
Condom	3	8.11	5	5.56	
Tubal resection	10	27.03	15	16.67	
Implantation	0	0.00	1	1.11	
Baseline Pap smear					0.253
WNL	1	2.70	1	1.11	
Inflammation	0	0.00	6	6.67	
ASCUS	6	16.22	18	20.00	
ASC-H	0	0.00	3	3.33	
LSIL	28	75.68	61	67.78	
HSIL	1	2.70	1	1.11	
SCCA	1	2.70	0	0.00	

WNL: within normal limit; ASCUS: atypical squamous cells of undetermined significance; ASC-H: atypical squamous cells, cannot rule out high-grade squamous intraepithelial lesion; LSIL: low-grade squamous intraepithelial lesion; HSIL: high-grade squamous intraepithelial lesion; SCCA: squamous cell carcinoma. * *p*-value < 0.05 indicated a statistically significant difference using the Fisher exact test.

**Table 2:** Univariable and multivariable analyses of the association between risk factors and regression status.

Characteristics	Univariable Analysis		Multivariable Analysis	
	HR (95% CI)	*p*-Value	HR (95% CI)	*p*-Value
HIV status	2.42 (1.26–4.61)	0.008 *	2.54 (1.31–4.94)	0.006 *
Age over 30 years	0.85 (0.39–1.87)	0.693	1.32 (0.51–3.42)	0.567
Parity				
1	0.58 (0.26–1.29)	0.181	0.51 (0.22–1.17)	0.111
2	0.52 (0.22–1.24)	0.141	0.41 (0.14–1.21)	0.107
3	0.12 (0.16–0.94)	0.044 *	0.10 (0.01–0.82)	0.032 *
≥4	1.20 (0.34–4.17)	0.777	0.94 (0.23–3.91)	0.932
Age of 1st SI				
20–29 years	0.68 (0.34–1.36)	0.271	0.60 (0.27–1.33)	0.207
30–39 years	1.19 (0.35–4.00)	0.783	0.87 (0.22–3.40)	0.839
≥40 years	1.83 (0.25–13.67)	0.555	1.24 (0.15–10.25)	0.840
Contraception used	0.77 (0.40–1.48)	0.428	0.83 (0.41–1.65)	0.587
Baseline Pap smear > LSIL	3.05 (0.73–12.74)	0.126	3.60 (0.74–17.50)	0.112

SI: sexual intercourse; LSIL: low-grade squamous intraepithelial lesion; HR: hazard ratio * *p*-value < 0.05 indicated a statistically significant association.

**Table 3:** Status of non-regression (persistent) disease and management.

Non-Regression Status/ Treatments	HIV-Positive (N = 19)	HIV-Negative (N = 18)	*p*-Value
Follow-up time of detection			0.082
- 6 months	12 (63.16%)	7 (38.89%)	
- 12 months	4 (21.05%)	2 (11.11%)	
- 18 months	2 (10.53%)	2 (11.11%)	
- 24 months	1 (5.26%)	7 (38.89%)	
Persistence of LSIL			NA
- Continue close follow-up	7 (36.84%)	9 (50.00%)	
- LEEP	3 (15.79%)	4 (22.22%)	
- TAH	1 (5.26%)	1 (5.56%)	
Progression			NA
- HSIL: LEEP	7 (36.84%)	4 (22.22%)	
- Cervical cancer stage IA1: TAH	1 (5.26%)	0 (0.00%)	

LEEP: loop electrosurgical excision procedure; TAH: total abdominal hysterectomy *p*-value < 0.05 indicated a statistical significance.

**Table 4:** Association between CD4 count and histological LSIL regression in women with HIV.

CD4 Count (Cells Per μL)	Total (N = 37)	Regression Status		*p*-Value
		Non-Regression (N = 15)	Complete Regression (N = 22)	
Mean ± SD		360.27 ± 207.85	589.95 ± 225.84	0.004 *
CD4 < 500 CD4 ≥ 500	20 (51.05%) 17 (45.95%)	11 (73.33%) 4 (26.67%)	9 (40.91%) 13 (59.09%)	0.053

* *p*-value < 0.05 indicated a statistical significance. SD: standard deviation.

## Data Availability

The raw data supporting the conclusions of this article will be made available by the authors upon request.

## Abbreviations

The following abbreviations are used in this manuscript:

LSILs	low-grade squamous intraepithelial lesions
CIN1	cervical intraepithelial neoplasia grade 1
HIV	human immunodeficiency virus
Pap smear	Papanicolaou smear
HSIL	high-grade squamous intraepithelial lesion
CDB	colposcopic directed biopsy
CR	complete regression
Para	parity (the number of times a patient has given birth to a viable child
SI	sexual intercourse
LEEP	loop electrosurgical excision procedure
WNL	within normal limit
ASCUS	atypical squamous cells of undetermined significance
ASC-H	atypical Squamous cells, cannot rule out high-grade squamous intraepithelial lesions
SCCA	squamous cell carcinoma
TAH	total abdominal hysterectomy
ASCCP	American Society for Colposcopy and Cervical Pathology
HPV	human papillomavirus
HR	hazard ratio
CD4	cluster of differentiation 4 (glycoproteins present on the immune cells)
Yrs	year
OCP	oral contraceptive pill
DMPA	depot medroxyprogesterone acetate (injected contraception)
TR	tubal resection (for contraception)
